# Supplementary material for: Comparative Genome Analysis of Japanese Field-Isolated Aspergillus for Aflatoxin Productivity and Non-Productivity
Source: J Fungi (Basel). 2024 Jun 28;10(7):459. doi: 10.3390/jof10070459 (PMC11278155; doi:10.3390/jof10070459)
Supplement: Supplementary file 1 [file jof-10-00459-s001.zip › jof-3058957-supplementary.pdf]

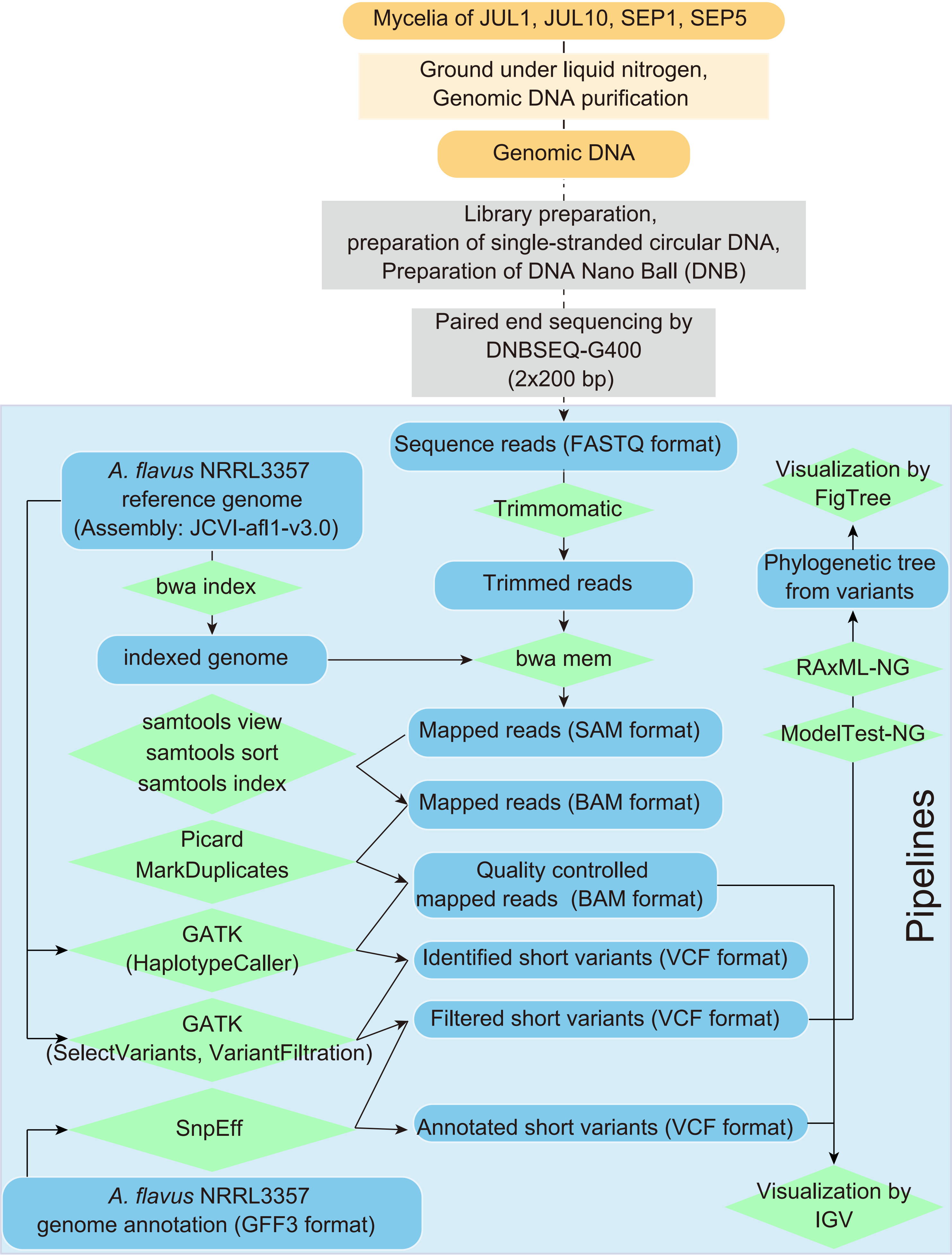

Figure S1. Sequencing data analysis pipeline in this study.

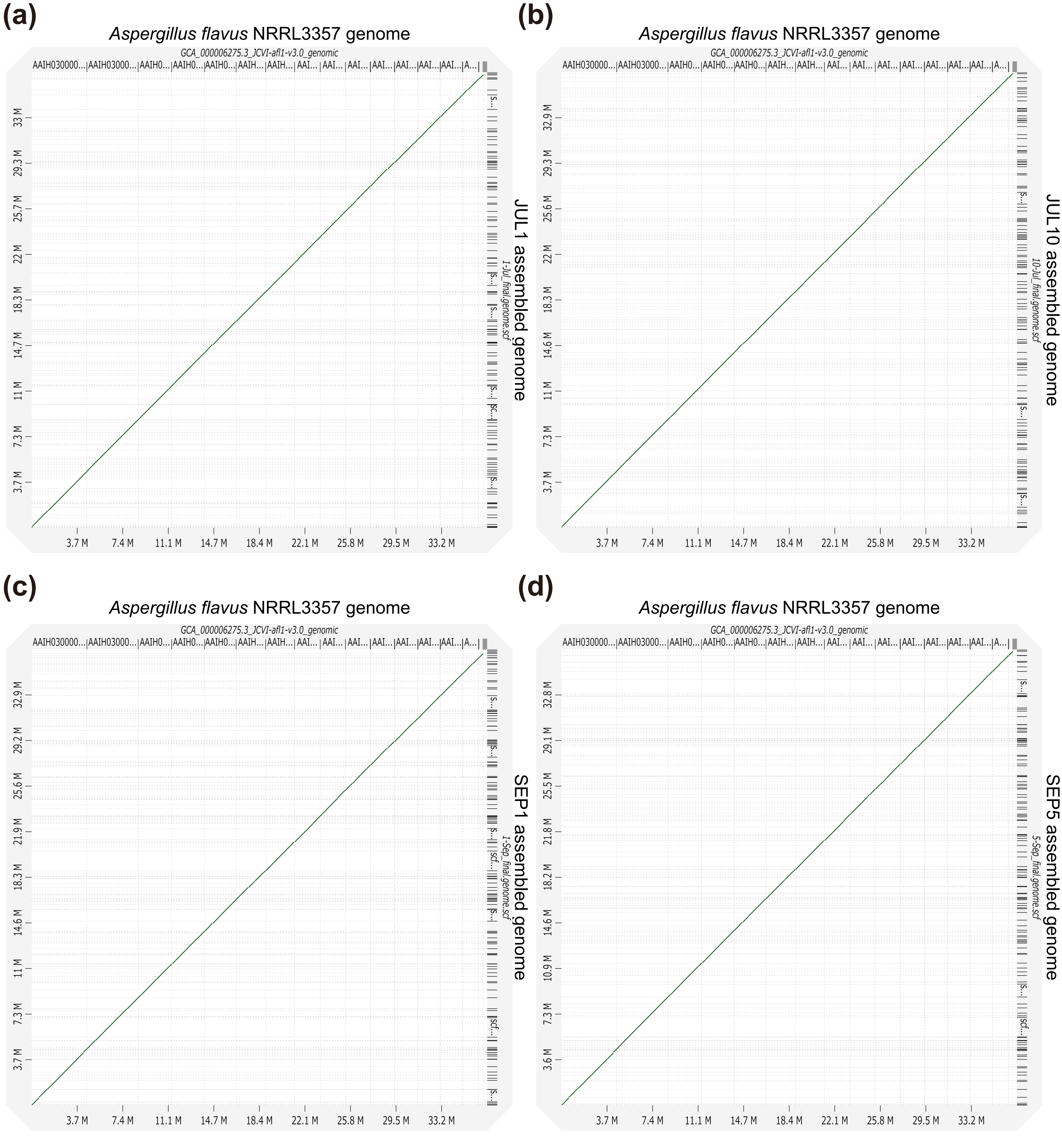

Figure S2. Dot plots comparing *A. flavus* NRRL3357 genome assembly and assembled contigs of 4 isolates. (a) JUL1. (b) JUL10. (c) SEP1. (d) SEP5. Plots were generated by D-GENIES online tool [16].

The dots indicate the genomic locations of sequences with close similarity.
In (a) to (d), apart from small gaps representing sequences present in only one of the genomes, the dots align along the central diagonal, indicating the two genomes are nearly identical.

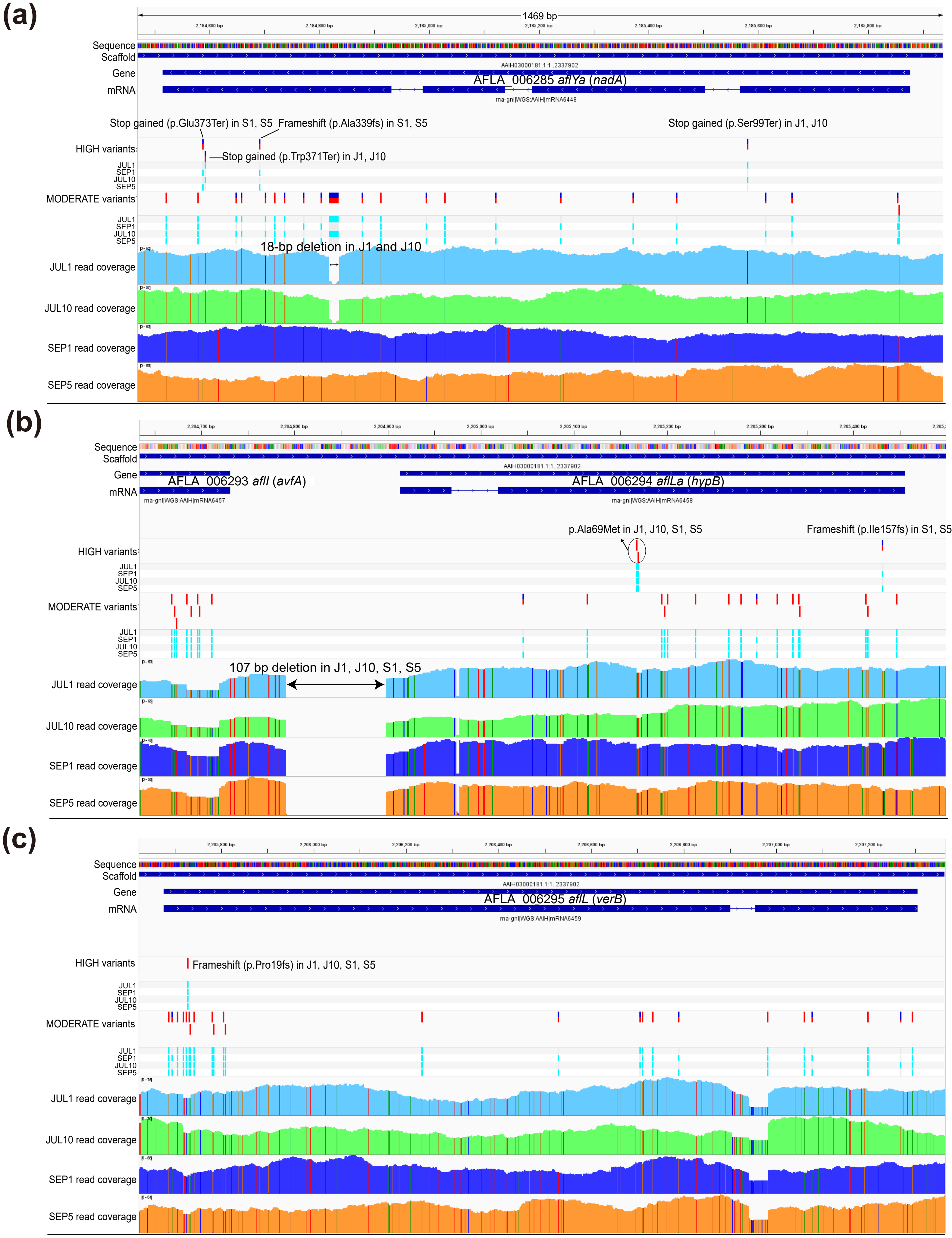

Figure S3. Genetic variants in the gene region of (**a**) *aflYa* (*nadA*), (**b**) *aflLa* (*hypB*), and (**c**) *aflL* (*verB*). J1, J10, S1, and S5 represents JUL1, JUL10, SEP1, and SEP5, respectively.
A. flavus NRRL3357 genome assembly JCVI-afl1-v3.0 was used as a reference.

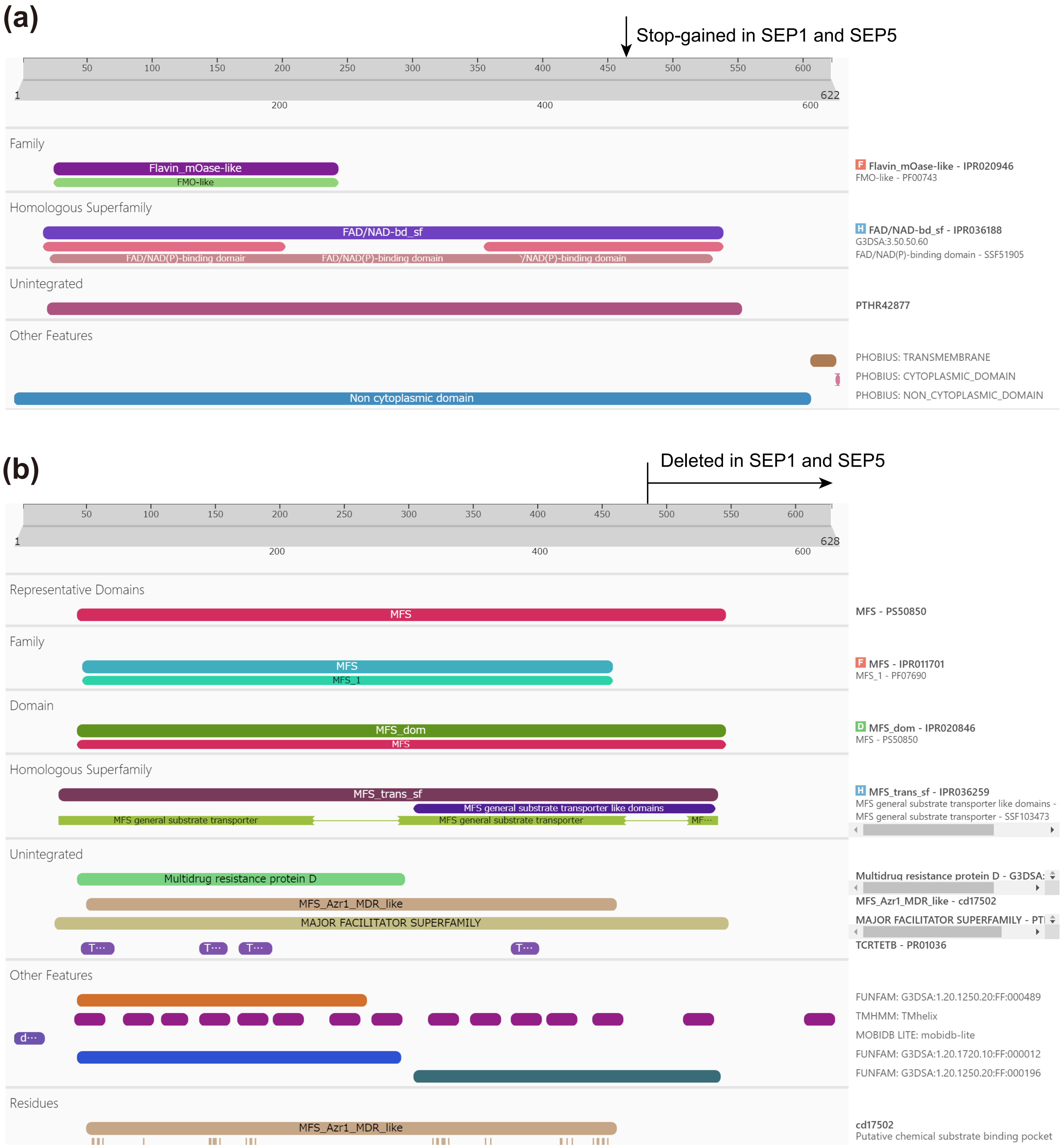

Figure S4. Domain search results of protein sequences of *aflW* (*moxY*) and *aflT* genes. Protein features and domains were predicted by InterProScan search for deduced protein sequences of *aflW* (*moxY*) (a) and *aflT* (b) genes.Arrows indicate the location of variants detected in SEP1 and SEP5.
